# The Charcot Marie Tooth disease protein LITAF is a zinc-binding monotopic membrane protein

**DOI:** 10.1042/BCJ20160657

**Published:** 2016-10-27

**Authors:** Wenxia Qin, Lydia Wunderley, Anne L. Barrett, Stephen High, Philip G. Woodman

**Affiliations:** Faculty of Biology Medicine and Health, University of Manchester, Manchester M13 9PT, U.K.

**Keywords:** CDIP1, endosome, ESCRT, membrane integration

## Abstract

LITAF (LPS-induced TNF-activating factor) is an endosome-associated integral membrane protein important for multivesicular body sorting. Several mutations in LITAF cause autosomal-dominant Charcot Marie Tooth disease type 1C. These mutations map to a highly conserved C-terminal region, termed the LITAF domain, which includes a 22 residue hydrophobic sequence and flanking cysteine-rich regions that contain peptide motifs found in zinc fingers. Although the LITAF domain is thought to be responsible for membrane integration, the membrane topology of LITAF has not been established. Here, we have investigated whether LITAF is a tail-anchored (TA) membrane-spanning protein or monotopic membrane protein. When translated *in vitro*, LITAF integrates poorly into ER-derived microsomes compared with Sec61β, a *bona fide* TA protein. Furthermore, introduction of N-linked glycosylation reporters shows that neither the N-terminal nor C-terminal domains of LITAF translocate into the ER lumen. Expression in cells of an LITAF construct containing C-terminal glycosylation sites confirms that LITAF is not a TA protein in cells. Finally, an immunofluorescence-based latency assay showed that both the N- and C-termini of LITAF are exposed to the cytoplasm. Recombinant LITAF contains 1 mol/mol zinc, while mutation of predicted zinc-binding residues disrupts LITAF membrane association. Hence, we conclude that LITAF is a monotopic membrane protein whose membrane integration is stabilised by a zinc finger. The related human protein, CDIP1 (cell death involved p53 target 1), displays identical membrane topology, suggesting that this mode of membrane integration is conserved in LITAF family proteins.

## Introduction

Cells communicate with their environment by the internalisation of lipids and membrane proteins from their surface. Once endocytic cargoes enter the early endosome, they are either sorted into recycling pathways [1–3] or trafficked towards the lysosome where they are degraded [4,5]. A central event in the degradative pathway is the formation of the multivesicular body (MVB) [6,7]. Here, degradative membrane cargoes are sorted to regions of the endosomal-limiting membrane that subsequently invaginate to form intralumenal vesicles. As intralumenal content accumulates, the MVB matures into a late endosome capable of fusing directly with the lysosome. Not all MVB-like compartments are destined for lysosomal degradation, however. For example, some MVBs fuse with the plasma membrane, thereby discharging their contents as secreted vesicles termed exosomes [8].

Many MVB cargoes utilise ubiquitination as a sorting signal [9,10], which is acted on by ESCRTs (endosomal sorting complexes required for transport) [7]. Upstream ESCRTs (ESCRTs-0, -I, and -II) bind ubiquitin and thus cluster MVB cargo within the endosome-limiting membrane [7]. ESCRT-III acts downstream to drive ILV formation [11,12]. Several ESCRT accessory proteins are also required for MVB sorting, including Bro1 proteins, ubiquitin ligases, and deubiquitinases [7,10,13–15].

One recently identified ESCRT accessory protein required for MVB sorting is LITAF (LPS-induced TNF-activating factor). LITAF (also known as SIMPLE; small integral membrane protein of the late endosome) is an endosome-associated membrane protein [16,17] that is important for ESCRT-dependent MVB sorting of activated epidermal growth factor receptor (EGFR) [18] and which also participates in exosome formation [19]. Consistent with a role in MVB sorting, LITAF interacts with the ESCRT-I component TSG101 (tumour susceptibility gene 101) [20] and ITCH/Nedd4 family ubiquitin ligases [17,20] via conserved N-terminal PSAP and PPXY peptide motifs. Underscoring the cellular importance of LITAF, several LITAF mutations cause autosomal-dominant Charcot Marie Tooth disease (CMT) type 1C (CMT1C), a demyelinating disorder of the peripheral nervous system [21,22]. Cells expressing LITAF bearing disease mutations are defective in MVB sorting [18,19]. LITAF may be important for other cellular events, since it is induced by bacterial toxins [23] and by p53 expression [24], and has been implicated in inflammatory diseases [25].

**Figure 1.**
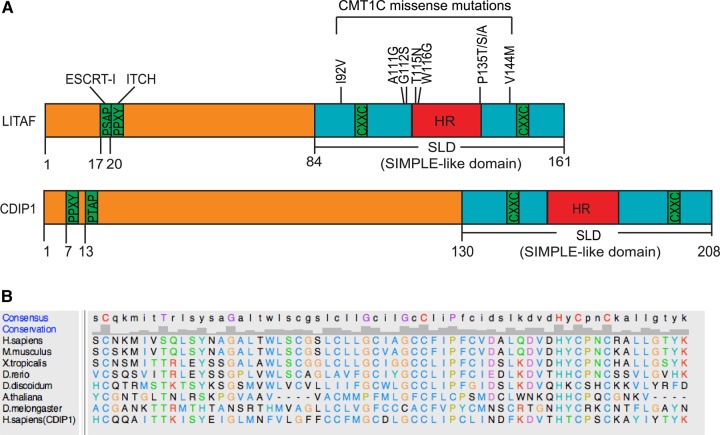
Domain organisation of LITAF family proteins. (**A**) Domain structures of LITAF and CDIP1. HR refers to the hydrophobic region. Binding motifs for ESCRT-I and ITCH/Nedd4 are shown. CMT1C disease mutations within LITAF are indicated. (**B**) Alignment of LITAF domains from selected eukaryotes. Sequences were obtained from Flybase, Dictybase, or Uniprot and aligned using Clustal Omega. UCSF Chimera was used to generate the figure using ClustalX colouring (http://www.jalview.org/help/html/colourSchemes/clustal.html). An identity histogram and consensus sequence are shown above.

A central feature of LITAF's domain organisation is a highly conserved C-terminal region known as the ‘LITAF domain’ or ‘SIMPLE-like domain’ (see Figure 1A). Here, a potential membrane-spanning 22 residue hydrophobic region is interposed between two cysteine-rich regions. The hydrophobic region is clearly crucial to the function of LITAF, since CMT mutations map closely to it. Despite this, the membrane topology of LITAF has yet to be determined, though differing models have been proposed [16,26,27] (see Figure 4A). Furthermore, given its high degree of conservation throughout eukaryotes (see Figure 1B), understanding the membrane topology of the LITAF domain has widespread importance. For example, mammalian cells express a related protein, CDIP1 (cell death involved p53 target 1), like LITAF possessing conserved PT/SAP and PPXY motifs and the C-terminal ‘SIMPLE-like’ domain (Figure 1A) [28]. Here, we show that LITAF defines a novel class of monotopic integral membrane proteins, in which the hydrophobic region is stably inserted into the membrane by virtue of a zinc-binding domain formed by the neighbouring cysteine-rich regions. This organisation is likely a general feature of LITAF domain proteins, since the same membrane topology is found in CDIP1.

## Experimental procedures

### Antibodies

TAT1 anti-tubulin was a gift from Keith Gull (University of Oxford). The following commercial antibodies were used. Mouse: anti-LITAF (Santa Cruz); anti-transferrin receptor (Zymed); anti-Strep-Tag (Novagen); anti-CD63 (Millipore); anti-EEA1 (early endosome antigen 1; BD Biosciences); anti-LAMP1 (lysosome-associated membrane protein 1; Developmental Studies Hybridoma Bank, University of Iowa); anti-OPG2 (opsin tag containing two glycosylation sites) was described previously [29]. Rabbit: anti-TorsinA was a gift from Lisa Swanton (University of Manchester); anti-V5 (Abcam); anti-EEA1, anti-LAMP1 (Cell Signaling). Sheep: anti-GFP was an in-house antibody generated against GST-GFP. Fluorescent secondary antibodies for IF and for Licor immunoblotting were from Jackson ImmunoResearch Laboratories (PA, USA).

### Molecular reagents

#### Mammalian constructs

Strep-LITAF was generated by cloning the LITAF ORF into the BamHI site of pTriEx5 (Novagen) to include a Strep-tag at the 5′-end of the sequence. For V5-CDIP1, V5-encoding oligos were first added between the EcoRV and XhoI sites of pcDNA5, followed by insertion of the CDIP1-coding sequence into the XhoI site. The TfR ORF was cloned into the EcoRV site of the above pcDNA5 vector to obtain TfR-V5. TfR-GFP has been described previously [30]. GFP-LITAF and GFP-CDIP1 were generated by inserting the LITAF or the CDIP1-coding sequence between the XhoI and BamHI sites of pEGFP-C1. LITAF-V5 and CDIP-V5 PCR products were sub-cloned into the same vector to obtain GFP-LITAF-V5 and GFP-CDIP1-V5. Sec61β-OPG2 and Cecropin-OPG2 were generated as described previously [31].

#### PCR product for *in vitro* translation experiments

The V5-CDIP1–Sec61β–OPG2 template used the forward primer: taatacgactcactataggGCAGATATCatgGGTAAGCCTATC, thereby introducing the T7 promotor. Other templates all used a standard CMV forward primer. The reverse primers used were:

Strep-LITAF TM-Sec61β-OPG2CT

5′-AGCTCCTGCGGCCGCTCAGTCTACTGTTTTGTTGCTGAATGGTACGTAGAAGTTTGGTCCTTCTGTTCCGTTCATTCTGCTCGAACGAGTGTACTTGAAGGGGATGAAGCAGCAGCC

Sec61β-LITAF-L155N

5′-CTACAAACGCTTGTAGGTGCCGTTGAGAGCTCTGCAGTTGGGACAGTAATGGTCCACGTCCTGCAGGGCATCCACGCCCCAAATGTGCAACATAAATAC

V5-CDIP1–Sec61β–OPG2CT

5′-AACCTCGAGTCAGTCTACTGTTTTGTTGCTGAATGGTACGTAGAAGTTTGGTCCTTCTGTTCCGTTCATTCTGCTCGAACGAGTGTACTTCAGGCAGCAGCCCAGATCACA

Sec61β-CDIP1-I201N

5′-TTAGCACAGGCGCTTGTACGTGTAGtTGTAGGCTTTGCAGCTGGGGCATGTGTGCGTCACATCCTTGAAGTCATTGATGAGGCAGGGGATCTTGCCCCAAATGTGCAACAT

### Cell culture and transfection

HeLa cells, or a derived cell line, HeLaM, were grown in DMEM supplemented with 1% NEAA, 10% foetal calf serum (HyClone; Perbio), and 1% Pen-Strep. Transient transfections were performed using JetPei (Polyplus) or Fugene6 (Roche). Transfection with siRNA was performed using Interferin (Polyplus). All siRNAs were ON-TARGETplus from Dharmacon. The LITAF siRNA oligo sequences were 08: 5′-UGCAGGACGUGGACCAUUA; 05: 5′-GCAUGAAUCCUCCUUGGUA. ON-TARGETplus Non-targeting was used as a negative control.

### Immunofluorescence and imaging

Cells were fixed in 3% formaldehyde in PBS and quenched with glycine, then permeabilised for 3 min in PBS containing 0.1% Triton X-100. Alternatively, cells were fixed in methanol at −20°C. For cell surface staining latency assays, antibodies were pre-incubated with cells in DMEM for 20 min at 4°C, and then cells were washed in PBS and fixed in 3% formaldehyde and quenched with glycine. Fluorescence was imaged on an Olympus BX60 upright microscope fitted with a 60 × 1.4 NA Plan Apo objective or a 100 × 1.3 NA Plan Apo objective and CoolSnap ES camera, and 12-bit images were captured using MetaVue software. For some experiments, cells were imaged by confocal microscopy using an Leica SP8 microscope. All images were opened as 16-bit grey-scale images and scaled using linear transformations in ImageJ, then converted to 24-bit RGB files in PhotoShop CS.

A non-biased, object-based method utilising ImageJ was used to determine the co-localisation of LITAF with endosomal markers. Wide-field images (each containing 4–5 cells) from three independent labelling experiments were superimposed on a grid (100-pixels area per grid unit) and LITAF-containing punctae that lay on grid lines were identified to obtain a random selection of these structures. Each LITAF punctum was then scored for whether it was positive for an endosomal marker. For EEA1 images, a total of 387 LITAF punctae were scored. For LAMP1 images, a total of 405 LITAF punctae were scored. Values are means ± SD from the three experiments.

### *In vitro* translations

Transcription was performed using T7 RNA Polymerase (Promega) according to the manufacturer's instructions, followed by purification with an RNeasy Mini kit (Qiagen). *In vitro* membrane translocation assays based on N-linked glycosylation have been described [29]. Translation reactions (25 µl) were carried out using nuclease-treated rabbit reticulocyte lysate (Promega) in the presence of EasyTag EXPRESS ^35^S Protein Labelling Mix containing [^35^S] methionine (PerkinElmer; 0.769 MBq; 43.5 TBq/mmol), 30 µM amino acids minus methionine (Promega), ∼1 µg of *in vitro* transcribed RNA, and 10% (v/v) nuclease-treated rough microsomes [optical density at 280 nm (OD280) = 44/ml]. The reaction was incubated at 30°C for 30 min to generate translation products and membranes were recovered through an 80 µl high-salt cushion [0.75 M sucrose, 0.5 M KOAc, 5 mM Mg(OAc)_2_, 50 mM HEPES–KOH, pH 7.9] at 100 000 ***g*** for 10 min at 4°C in a TLA100 rotor (Beckman). The membrane pellet was resuspended in 20 µl low-salt buffer [100 mM sucrose, 100 mM KOAc, 5 mM Mg(OAc)_2_, 50 mM HEPES–KOH, pH 7.9, 1 mM DTT] and treated with 250 µg/ml RNase A at 37°C for 10 min to remove any residual tRNA species. For carbonate extraction, recovered membranes were incubated with 0.1 M Na_2_CO_3_ (pH 11) on ice for 15 min, followed by centrifugation at 100 000 ***g*** for 10 min at 4°C in a TLA100 rotor (Beckman). For endoglycosidase H (EndoH) treatment, 1000 IU Endo Hf (NEB) was incubated with samples in SDS–PAGE buffer at 37°C for 2 h

### Cell-based biochemistry

For carbonate extraction experiments, samples were incubated with 0.1 M Na_2_CO_3_ (pH 11) on ice for 15 min, followed by centrifugation at 100 000 ***g*** for 10 min. The supernatant was discarded and the pellet was resuspended in sample buffer. For immunoprecipitations, cells were lysed in IP buffer [20 mM Tris–HCl (pH 7.4), 100 mM NaCl, and 1% Triton X-100] containing Protease Inhibitor Cocktail-III (Sigma). Lysates were clarified at 14 000 rpm for 15 min at 4°C and incubated with antibodies at 4°C overnight, then 3–4 h with protein A sepharose (Zymed), preblocked with 50 mg/ml BSA. Beads were washed four times in IP buffer, then analysed by Western blot (WB). Control IPs used non-immune sera or IgGs. For EndoH assays, samples were treated as for *in vitro* translation experiments. For PNGase assays, cell lysates were subjected to immunoprecipitation, followed by incubation with 500 IU PNGase F (NEB) at 37°C for 2 h prior to WB analysis.

### Expression and purification of recombinant proteins for zinc analysis

For zinc analysis, LITAF or Sec61β was sub-cloned into pMAL-c5X (NEB). Proteins were expressed in *Escherichia coli* strain BL21 (DE3) pLysS by IPTG induction, including 1% glucose and 100 μM zinc chloride. Cell pellets were resuspended in 20 mM Tris pH 7.4, 200 mM NaCl supplemented with 1 mM PMSF, complete protease inhibitor cocktail (Roche), Benzonase, and 0.5% lauryldimethylamine-*N*-oxide (LDAO). The cells were lysed by sonication and insoluble material was pelleted at 17 000 ***g*** for 60 min at 4°C. Amylose resin (NEB) was used to isolate the recombinant protein. Elution from the resin was performed using 10 mM maltose. Further purification was performed by size-exclusion chromatography in 10 mM Tris pH 7.4, 150 mM NaCl, and 0.5% LDAO. Metal analysis by inductively coupled plasma mass spectrometry (ICP-MS) was performed by Dr Heinrich Baumann (Spurenanalytisches Laboratorium, Maxhütte-Haidhof).

## Results

### LITAF and CDIP1 localise to endocytic compartments

**Figure 2.**
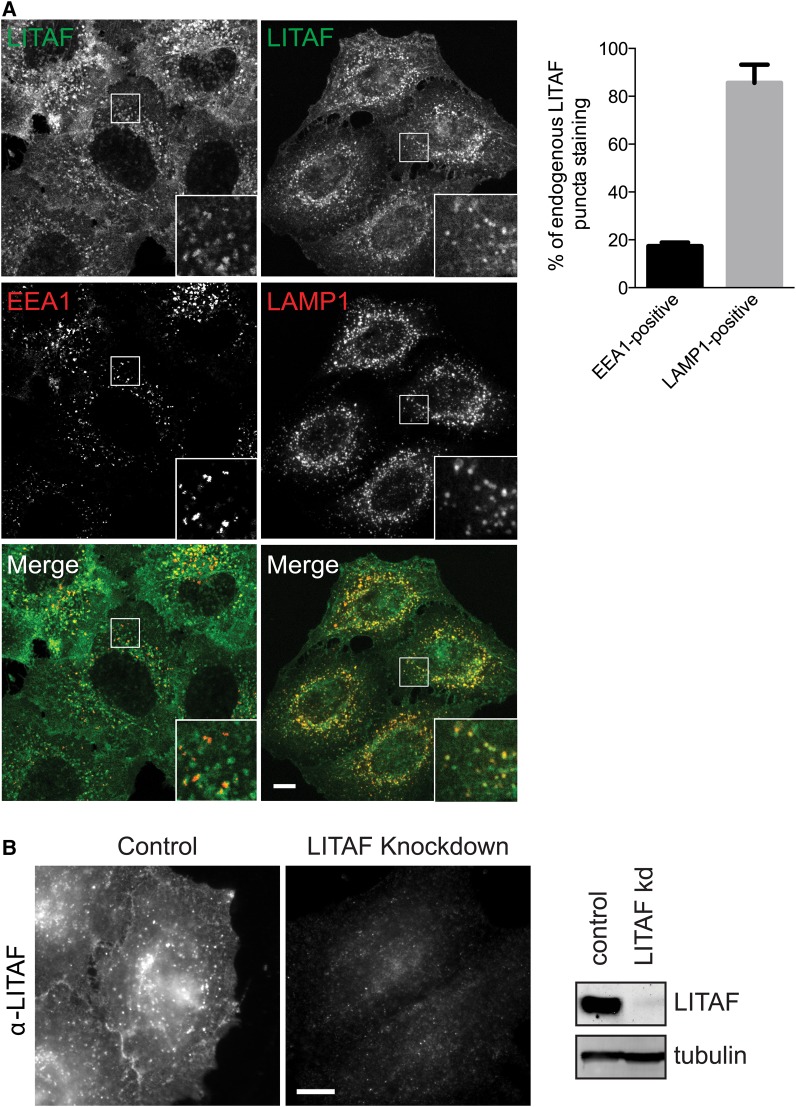
LITAF localisation. (**A**) IF of endogenous LITAF vs. EEA1 or LAMP1 in HeLaM cells. IF is by confocal microscopy. Bar chart shows colocalisation of LITAF with either LAMP1 or EEA1. Data are averaged from three independent experiments ± SD. A total of 12 cells were analysed for EEA1 and 14 cells were analysed for LAMP1. (**B**) HeLaM cells were transfected with control or LITAF siRNA and immunostained or Western blotted for LITAF. IF is by wide-field microscopy. Scale bars = 10 µm. Insets magnified ×3.

Previous studies differ in their description of LITAF (Figure 1A) as a late or an early endosomal protein [16–20]. We therefore co-stained HeLaM cells for endogenous LITAF vs. early and late endosomal compartment markers. Endogenous LITAF localised partially to EEA1-positive early endosomes and more extensively to LAMP1-positive late endosomes/lysosomes (Figure 2A). Quantitation showed that <20% of LITAF punctae co-localised with EEA1, while ∼85% co-localised with LAMP1. Endogenous LITAF was also found at the plasma membrane (Figure 2A,B). Control experiments using LITAF siRNA confirmed that the LITAF staining was specific (Figure 2B). Hence, LITAF localises to the cell surface and to several endocytic compartments.

**Figure 3.**
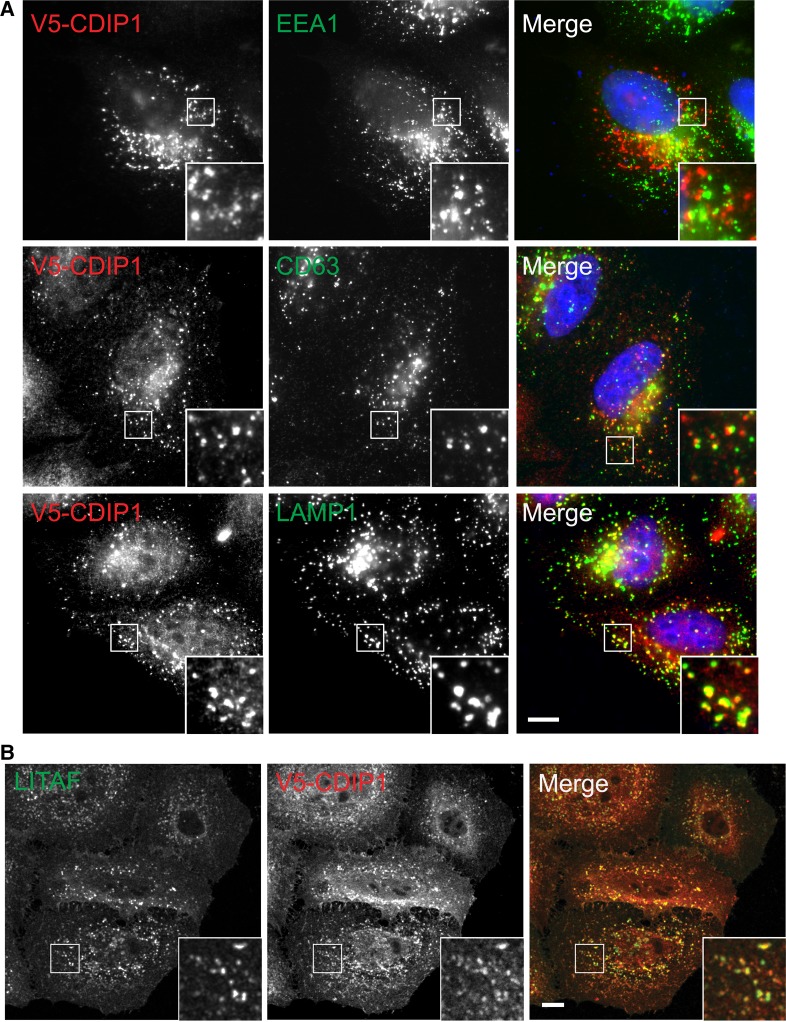
CDIP1 localises to the endocytic pathway. (**A**) HeLaM cells expressing V5-CDIP1 were analysed by IF with anti-V5 vs. the indicated endocytic markers. IF is by wide-field microscopy. (**B**) HeLaM cells expressing V5-CDIP1 were analysed by IF with anti-V5 and anti-LITAF. IF is by confocal microscopy. Scale bars = 10 µm. Insets magnified ×3.

We then examined the localisation of CDIP1, which is related to LITAF (Figure 1B) and which has previously been implicated in ER function [32]. Though we found no anti-CDIP1 antibody that detected the endogenous protein by IF, epitope-tagged CDIP1 localised predominantly to CD63-positive late endosomes as well as to LAMP1-positive compartments, but not clearly to early endosomes (Figure 3A). A minor pool of CDIP1 could also be detected on the cell surface. Epitope-tagged CDIP1 also showed substantial co-localisation with endogenous LITAF (Figure 3B). Hence, CDIP1 also localises to the endocytic pathway, although at steady state it may be present in somewhat later compartments than LITAF. Although we could not detect CDIP1 staining at the ER, our findings do not preclude separate functions for CDIP1 at the ER, as reported previously [32], that could be attributable to a small or transient pool of the protein.

**Figure 4.**
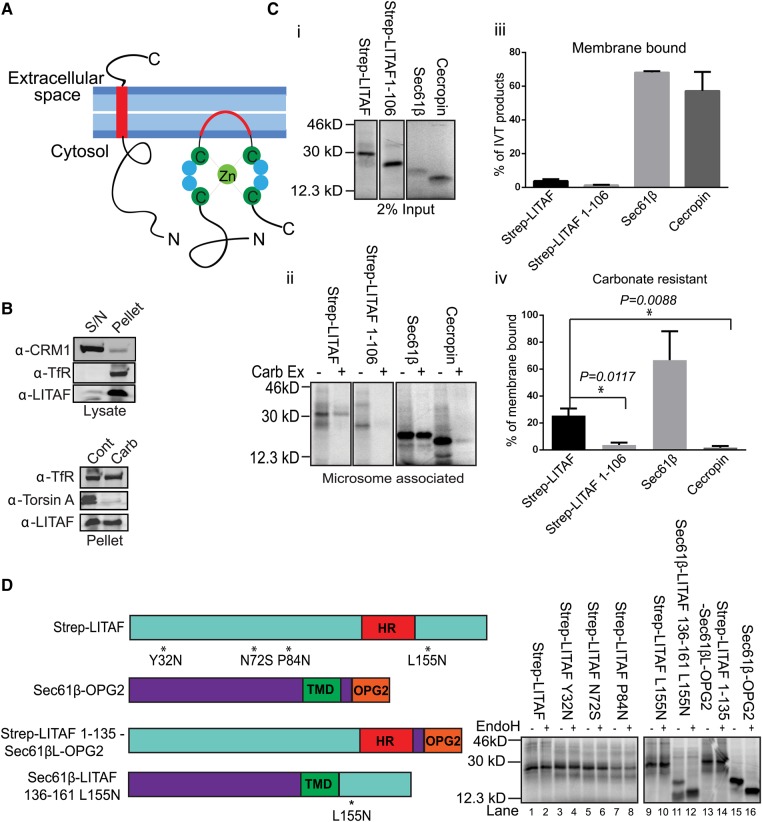
LITAF does not behave as a TA membrane protein in an *in vitro* system. (**A**) Alternative models for LITAF membrane integration. (**B**) Top panel: whole-cell lysates from HeLaM cells were separated into cytosol (S/N) and membrane (pellet) fractions, and blotted for LITAF, the soluble nuclear export factor CRM1, or transferrin receptor (TfR). Bottom panel: membrane pellet fractions were treated with control buffer (cont) or 100 mM sodium carbonate pH 11.5 (carb), then re-pelleted, and immunoblotted for TfR, torsin A (a peripheral protein of the ER lumen), or LITAF. (**C**) (i) The indicated proteins were *in vitro* translated in the presence of ER membranes. (ii) Membranes were re-isolated, then washed in control buffer or 100 mM sodium carbonate pH 11.5 (Carb Ex). Samples were analysed by SDS–PAGE and phospho-imaging. (iii and iv) Showing the quantification of membrane-binding efficiency and carbonate resistance. Mean values from three independent experiments ± SD and statistical significance was tested using the Student's *t*-test. * denotes statistical significance (i.e. *P* < 0.05). (**D**) Strep-LITAF containing introduced glycosylation sites as indicated, Sec61β-OPG2, or LITAF/Sec61β chimaeras (left panel) were *in vitro* translated with ER microsomes. Re-isolated membranes were treated with or without EndoH (right panel).

### LITAF and CDIP1 are not tail-anchored membrane proteins

It has been proposed that LITAF (Figure 1A) is a tail-anchored (TA) integral membrane protein [26], but this membrane topology has not been verified experimentally. Bioinformatics analysis of Drosophila LITAF-like proteins [27] suggests that the LITAF C-terminal region might instead comprise a Zn^2+^ finger encompassing a hydrophobic sequence that would insert into the membrane but not translocate across it (Figure 4A). In such a scenario, several disease-linked mutations [21,22,33,34] would map close to or at the predicted membrane–cytosol interfaces of the hydrophobic insertion (Figure 1A).

Confirming a previous report [26], we found that endogenous LITAF is both membrane-associated and resistant to alkaline sodium carbonate extraction, consistent with it behaving as an integral membrane protein (Figure 4B). However, to test directly whether LITAF is indeed a TA protein, we performed *in vitro* translations in the presence of ER microsomes, since the TA proteins found within the endomembrane system are membrane integrated at the ER [35]. Although a fraction of *in vitro*-translated LITAF associated with microsomes, membrane targeting was much less efficient than for Sec61β, a TA protein, or cecropin, a secreted protein that translocates into the ER lumen (Figure 4C). The weak membrane association of LITAF required its C-terminal region, since LITAF (1–106) bound microsomes even less well than FL (Full length) LITAF (Figure 4C, panels i and iii). Furthermore, when the nature of the association between LITAF and microsomes was analysed further, only 20% of the microsome-associated LITAF was resistant to carbonate extraction, far lower than a *bona fide* TA protein, Sec61β (Figure 4C, panels ii and iv). As expected, the lumenal protein cecropin was completely extracted by carbonate. In summary, LITAF associates with ER microsomes, but less well than established substrates for ER translocation and hence may not be a TA protein.

To test the membrane topology of the pool of LITAF that associates with ER membranes, N-linked glycosylation sites were introduced at various positions, including on both sides of the hydrophobic region (HR) proposed [26] to act as a transmembrane domain (TMD; Figure 4D, left panel). Exposure of these sites to the ER lumen should generate higher MW form(s) of LITAF that are sensitive to endoglycosidase H (EndoH). These constructs were translated and then ER microsomes were re-isolated to allow the membrane-bound pool of LITAF to be analysed. None of these microsome-associated LITAF constructs were N-glycosylated (Figure 4D; lanes 1–10). In contrast, a Sec61β construct containing a glycosylation tag derived from bovine opsin (Sec61β-OPG2) was efficiently glycosylated (Figure 4D, lanes 15–16). A chimaera of the cytoplasmic and hydrophobic regions of LITAF (LITAF_1–135_) fused to the lumenal domain of Sec61β-OPG2 (LITAF_1–135_-Sec61β_L_-OPG2) was not glycosylated (Figure 4D, lanes 13 and 14). In contrast, a chimaera of the cytoplasmic and TM domains of Sec61β fused to LITAF_136–161_ and containing a glycosylation site (Sec61β-LITAF_136–161_L^155^N) was glycosylated (Figure 4D, lanes 11–12). Overall, these data show that the ER-bound pool of *in vitro*-translated LITAF does not span the membrane, behaviour quite distinct from Sec61β, a known TA protein.

**Figure 5.**
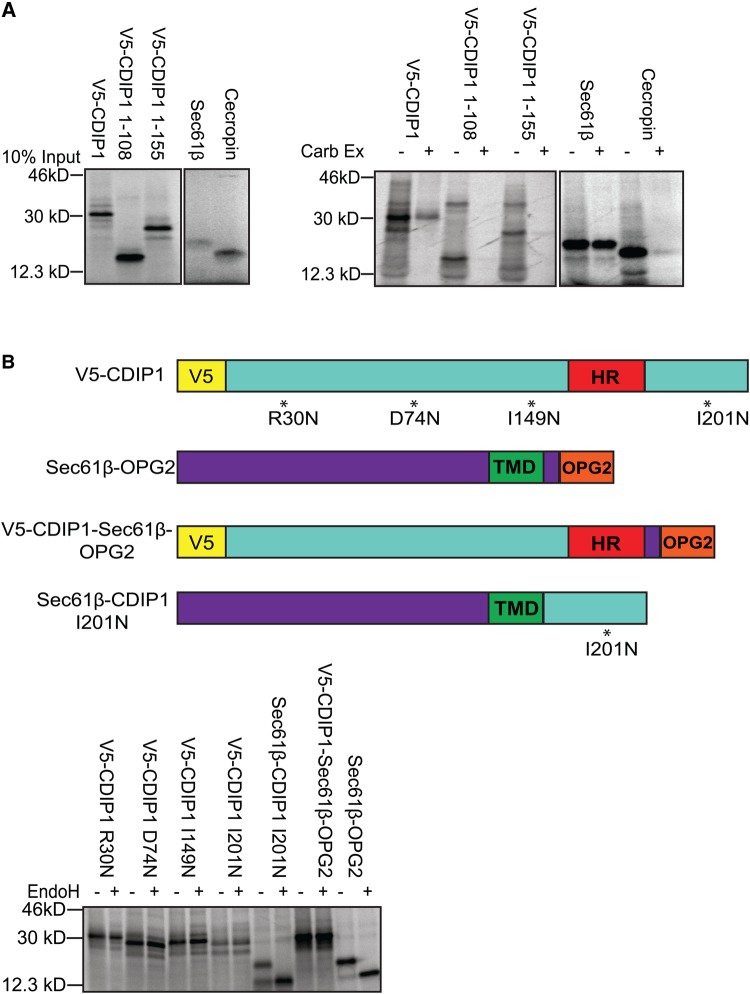
CDIP1 does not behave as a TA membrane protein. (**A**) Full-length V5-CDIP1, V5-CDIP1 truncations as indicated, Sec61β, or cecropin was *in vitro* translated in the presence of ER membranes (left panel). Membranes were re-isolated, washed in control buffer or 100 mM sodium carbonate pH 11.5 (Carb Ex), then analysed by SDS–PAGE, and phosphor-imaging (right panel). (**B**) V5-CDIP1 containing introduced glycosylation sites as indicated, Sec61β-OPG2, or CDIP1/Sec61β chimaeras (top panel for models) were *in vitro* translated with ER microsomes. Microsomes were re-isolated and treated with or without EndoH before being analysed by SDS–PAGE and phosphor-imaging (bottom panel).

Since the sequence organisation of CDIP1 resembles that of LITAF, most notably in the conserved C-terminal region responsible for membrane integration (Figure 1A), we also examined the ability of CDIP1 to integrate into microsomes. CDIP1 associated with, and inserted into, ER membranes with similar efficiency to LITAF (Figure 5A). As for LITAF, membrane association and integration were reduced by truncations that removed the C-terminal hydrophobic region. Moreover, CDIP1 bearing artificially inserted glycosylation sites, and CDIP1–Sec61β chimaeras, behaved precisely as LITAF constructs during *in vitro* translocation/glycosylation studies, displaying no evidence for the translocation of the C-terminus of the protein into the ER lumen (Figure 5B). The behaviour we describe is thus likely to be a general feature of proteins containing the LITAF domain.

**Figure 6.**
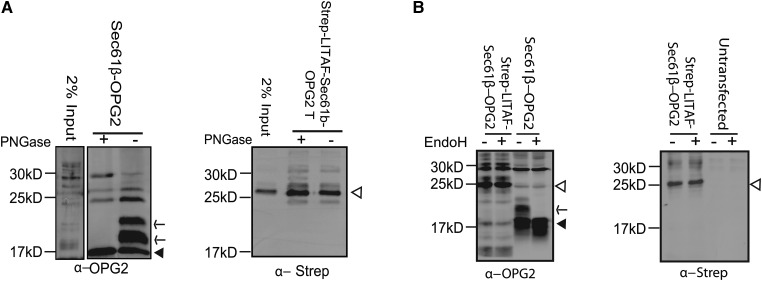
LITAF is not a TA protein in cells. (**A**) Lysates from HeLaM cells transfected with Sec61β-OPG2 (left panel) or with LITAF_1–135_-Sec61β_L_-OPG2 (right panel) were immunoprecipitated with anti-OPG2. Samples were treated with or without PNGaseF and then immunoblotted with anti-OPG2 (left panel) or anti-Strep-tag (right panel). Arrowheads indicate non-glycosylated products. Arrows indicate glycosylated Sec61β-OPG2. (**B**) Left panel: Lysates from HeLaM cells transfected with Sec61β-OPG2 or strep-LITAF_1–135_-β_lumen_-OPG2 were treated with or without EndoH and immunoblotted with anti-OPG2. Closed arrowhead denotes Sec61β-OPG2 and arrow denotes glycosylation product. Open arrow denotes LITAF_1–135_-β_lumen_-OPG2. Right panel: to further confirm the identity of LITAF-specific products, extracts from untransfected cells or cells transfected with strep-LITAF_1–135_-β_lumen_-OPG2 were treated with or without EndoH and immunoblotted with anti-Strep-tag. Open arrow denotes LITAF chimaera.

To test whether or not LITAF behaves like an ER-inserted TA protein in cells, LITAF_1–135_-Sec61β_L_-OPG2 was expressed and analysed for glycosylation based on sensitivity to the broad-spectrum glycosidase, PNGaseF (Figure 6A), or to EndoH (Figure 6B). EndoH cleaves the high-mannose sugars found in ER-localised glycoproteins, whereas PNGaseF also acts on Golgi-modified glycoproteins. The control protein, Sec61β-OPG2, was efficiently glycosylated as expected (Figure 6A,B). In contrast, no glycosylation of LITAF_1–135_-Sec61β_L_-OPG2 was detected (Figure 6A,B). Hence, LITAF does not behave as an ER-inserted TA membrane protein in cells.

### LITAF is a zinc-binding monotopic membrane protein

**Figure 7.**
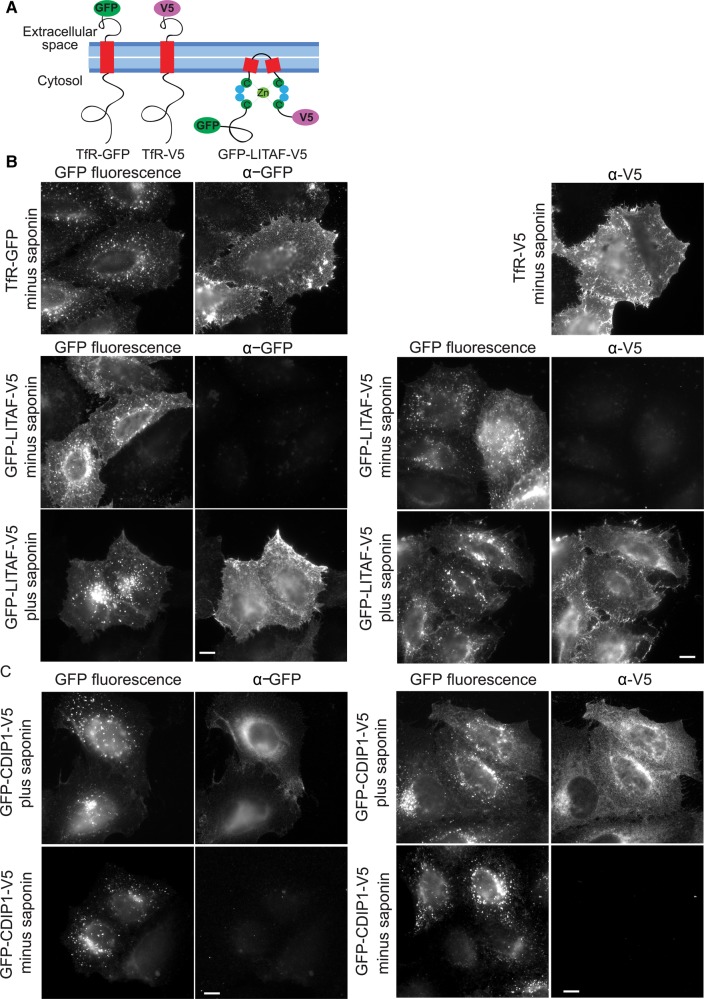
LITAF is a monotopic membrane protein. (**A**) Cartoon showing the positioning of epitope tags. (**B**) HeLaM cells were transfected with TfR-GFP, TfR-V5, or GFP-LITAF-V5, as indicated. Cells were incubated with primary antibodies at 4°C prior to fixation or incubated with antibody after they had been fixed and permeabilised with saponin. Scale bars = 10 µm. (**C**) HeLaM cells were transfected with GFP-CDIP1-V5 and processed as for IF with or without saponin permeabilisation.

To directly examine the membrane topology of LITAF in cells, GFP-LITAF-V5 (Figure 7A) was expressed and the membrane orientation of the cell surface pool was tested by performing IF with or without detergent. In a control experiment, anti-GFP or anti-V5 antibodies readily detected surface pools of TfR-GFP and TfR-V5, containing extracellular epitopes (Figure 7B). In contrast, neither antibody could detect surface GFP-LITAF-V5 without detergent present, but did so readily when cells were permeabilised with saponin (Figure 7B). Note that the intracellular pool of GFP-LITAF-V5 stained relatively weakly with anti-GFP or anti-V5 under these conditions. This is consistent with a significant pool of endosomal LITAF locating to ILVs [19], and hence not being accessible to antibodies even in the presence of saponin. In summary, LITAF is a monotopic membrane protein with both termini exposed to the cytoplasm. Similar experiments showed that CDIP1 has the same membrane topology (Figure 7C).

**Figure 8.**
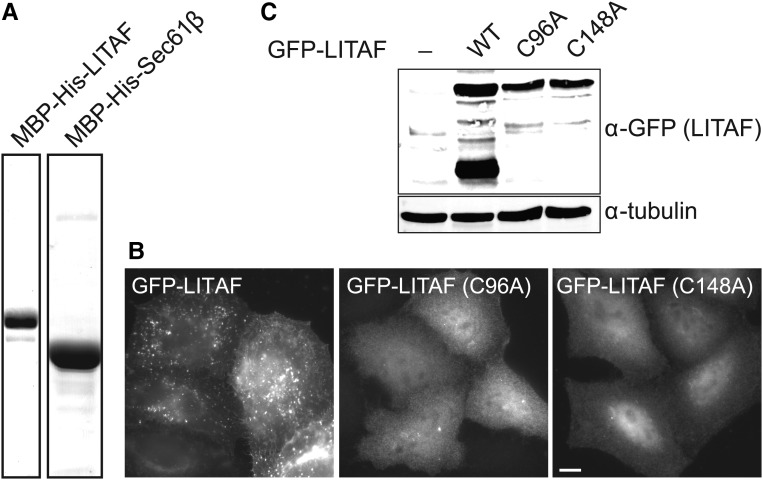
Membrane association of LITAF involves the LITAF domain zinc finger. (**A**) Recombinant LITAF or Sec61β was purified from bacterial lysates and analysed for zinc content by ICP-MS. (**B**) Fluorescence microscopy images of cells expressing GFP-LITAF mutants, as indicated. Wide-field microscopy. Scale bar = 10 µm. (**C**) HeLaM cells were transfected with WT GFP-LITAF, or the indicated mutants, and lysates were subjected to immunoblotting.

The LITAF C-terminal region has been postulated to include a Zn^2+^ finger, since it contains conserved motifs that match ligand-binding knuckles (CCPSC^99^ and HYCPNC^151^; Figure 1). Consistent with a single zinc-binding motif, ICP-MS on recombinant protein, purified to homogeneity (Figure 8A), showed that LITAF preparations contained 0.78 moles zinc/mole protein (±0.21 SD, *n* = 3), whereas zinc was not detected above background levels in Sec61β preparations. To test whether the cysteine motifs are important for the membrane integration of LITAF, C^96^A or C^148^A mutations were introduced into the GFP-tagged protein. These mutations completely prevented the membrane association of LITAF, generating a distinct cytoplasmic/nuclear localisation (Figure 8B). Immunoblotting controls showed that these mutants expressed properly and were not degraded (Figure 8C). Interestingly, the WT protein but neither mutant was subject to partial cleavage, suggesting that endosomal association might lead to proteolysis. In summary, the LITAF Zn^2+^ finger is important for stable membrane integration, further underscoring the membrane topology of the protein.

## Discussion

Here, we identify LITAF and CDIP1 as belonging to a novel, conserved class of monotopic integral membrane proteins that localise to the plasma membrane and endocytic compartments. Our experimentally determined membrane topology of LITAF contrasts with the proposal that LITAF is a TA protein possessing a classical membrane-spanning helix [26], but fully supports bioinformatics analysis suggesting that both the N- and C-terminal cysteine-rich regions within the LITAF domain are exposed to the cytoplasm [27]. Moreover, we provide evidence that these cysteine-rich regions combine to form a zinc finger necessary for stable insertion of the LITAF domain into membranes, as also proposed by Ponting et al. [27].

At present, we can only speculate on the conformation of the ∼22 amino acid hydrophobic regions of LITAF and CDIP1 predicted to insert into the membrane. It is possible that these form helices that insert into the cytoplasmic leaflet of the membrane in a manner analogous to the shorter amphipathic helices of ENTH domain proteins [36]. Alternatively, they may adopt hairpin-like configurations, analogous to the longer hairpin structures found in other monotopic proteins such as caveolin [37] or the double hairpin reticulon proteins [38,39]. In either respect, it is possible that one consequence of the asymmetrical insertion of LITAF into the lipid bilayer contributes to the induction or sensing of membrane curvature, and thus would represent a key event underpinning the endocytic trafficking functions of LITAF.

The LITAF membrane topology that we describe allows a rational basis for understanding the impact of known CMT1C-linked mutations. Virtually all of these mutations lie within the LITAF domain, within or either side of the core hydrophobic region [21,22,33,34]. Based on a monotopic membrane topology, these mutations would lie at, or very close to, the membrane–cytosol interface. It is likely, therefore, that the gain-of-function effects evident in CMT1C are caused by alterations in the structure of LITAF at this interface. These, in turn, might alter local lipid dynamics or prevent LITAF from binding additional trafficking factors at the membrane–cytosol interface, thereby interfering with endocytic trafficking. It is also possible that they could impact on how tightly LITAF is associated with the membrane. Indeed, several disease mutations have been reported to influence the membrane association of LITAF [26], though the mechanistic rationale for these findings has to date been unclear.

The unambiguous description of LITAF's membrane topology presented here is also consistent with other molecular studies. For example, peptide motifs close to the C-terminus of LITAF, which may act as AP2 clathrin adaptor-binding sites, appear essential for LITAF function during exosome formation [19]. The importance of these motifs is difficult to reconcile with a tail anchor model for LITAF membrane integration, since in this configuration the motifs would be exposed in the endosome lumen. Furthermore, the *Arabidopsis* LITAF protein GILP possesses both N- and C-terminal binding sites for its interaction with the cytosolic programmed cell death protein, LSD1 [40].

In summary, the identification of the molecular basis for the membrane integration of LITAF provides a new focus towards understanding the disease mechanisms underlying LITAF mutations and provides new insights into the contribution that LITAF makes to receptor trafficking on the endocytic pathway.

## Abbreviations

CDIP1: cell death involved p53 target 1
CMT1C: Charcot Marie Tooth disease type 1C
EEA1: early endosome antigen 1
EndoH: endoglycosidase H
ER: endoplasmic reticulum
ESCRT: endosomal sorting complex required for transport
GILP: GSH-induced LITAF domain protein
HR: hydrophobic region
ICP-MS: inductively coupled plasma mass spectrometry
IF: immunofluorescence
ILV: intralumenal vesicle
LAMP1: lysosome-associated membrane protein 1
LDAO: lauryldimethylamine-*N*-oxide
LITAF: LPS-induced TNF-activating factor
LPS: lipopolysaccharide
MVB: multivesicular body
MW: molecular weight
NEAA: non essential amino acids
OPG2: opsin tag containing two glycosylation sites
SIMPLE: small integral membrane protein of the late endosome
TA: tail-anchored
TSG101: tumour susceptibility gene 101
WB: Western blot.
